# Widening the Access to HIV Testing: The Contribution of Three In-Pharmacy Testing Programmes in Spain

**DOI:** 10.1371/journal.pone.0134631

**Published:** 2015-08-06

**Authors:** Sonia Fernández-Balbuena, María José Belza, Daniel Zulaica, Jose Luis Martinez, Henar Marcos, Benet Rifá, Arantxa Arrillaga, Luis de la Fuente, Juan Hoyos, Working Group

**Affiliations:** 1 National School of Health, Carlos III Health Institute, Madrid, Spain; 2 Consortium for Biomedical Research in Epidemiology & Public Health (CIBERESP), Madrid, Spain; 3 Plan for prevention and control of AIDS and STIs, Basque health service, San Sebastian, Spain; 4 Section of surveillance, prevention and control of STIs and HIV, Office of surveillance and response to public health emergencies, Public health agency of Catalonia, Department of health of the regional government of Catalonia, Barcelona, Spain; 5 Epidemiological Surveillance Service, Public Health Directorate, Regional Ministry of Health of Castilla and León, Valladolid, Spain; 6 National Epidemiology Centre, Carlos III Health Institute, Madrid, Spain; David Geffen School of Medicine at UCLA, UNITED STATES

## Abstract

**Background and Objective:**

Spain has implemented several in-pharmacy HIV testing programmes performed by pharmacists as part of their everyday routine. We aim to assess the feasibility and the main outcomes of three programmes implemented in three Spanish regions with different sociological profiles and also different epidemiology for HIV.

**Methods:**

The characteristics of the 24151 people tested between 2009 and 2013 at 74 urban pharmacies are studied. We compare the main outcomes of the programmes with those of each Regional HIV Surveillance System (RHSS) assessing the contribution to the total new diagnosis in each region and if priority groups are being reached.

**Results:**

45.7% were heterosexual men (MSW), 14.4% men who have sex with men (MSM), and 27% women. The 35% were younger than 30 and 9.6% foreigners. The 52% were previously untested, and women were the most likely to be untested. The three programmes altogether diagnosed 226 people, resulting in a global prevalence of 0.9% (95%CI: 0.8–1.1); 3.4% in MSM (95%CI: 2.8–4.0). The prevalence among Spaniards was 0.8% (0.7–1.0) vs. 2.2 (1.6–2.9) among foreigners. The percentages of MSM diagnosed by all three programmes were higher than the one reported by their respective RHSS. Thirty four percent of the reactive MSM and the 71.4% of the reactive MSW did not have a previous HIV test although big testing history differences were observed across the programmes. Altogether, these services contributed with the 10.6% of all HIV diagnoses in these regions.

**Conclusions:**

In-pharmacy HIV testing programmes are a valuable testing option, having been able to uncover 1 out of 10 the new diagnoses reported in each region. They showed a good capacity of reaching and diagnosing previously untested populations, not only a priority population such as MSM but also heterosexual population who are more affected by delayed diagnosis. They seem to be particularly suitable for regions without large cities and specific HIV diagnostic services.

## Background

HIV testing is the linchpin for prevention and treatment. The importance of early diagnosis from a patient, public health and cost perspective has been widely described. [1–4] In order to facilitate the access to HIV testing and control the epidemic, numerous strategies have been set up including the expansion of testing to community services and healthcare settings outside specialist services. [5–8]

In this regard, pharmacies have been considered as a novel strategy to make HIV testing more accessible to the general population.[9–11] Although they are a very low threshold services, operated by highly trained health professionals with a very favourable attitude towards providing HIV testing and counselling [12] they have been underused [13]. There are some published examples of pharmacy-provided HIV testing, but their scopes have always been very limited. An initiative in New York that used a counsellor to conduct HIV testing in 5 pharmacies in high HIV-prevalence areas, proved to be feasible. [14] However, the use of third-party partners to conduct the test raised sustainability concerns. Others relied on pharmacy staff to perform testing: in 2011, during the 3 days leading up to the National HIV Testing Day, the CDC partnered with Walgreens to offer free HIV testing at 22 stores, screening over 900 customers.[15] A pilot programme launched by the CDC in 2012 involving 21 sites (18 were community pharmacies) conducted 1087 tests [16]. Nevertheless, it is not clear if any of the participating pharmacies have continued offering the service once the pilot was over. Finally, the last example we have found is a programme that included only two pharmacies in Michigan and that tested 69 people in two years uncovering one infection.[17]

Spain has been a pioneer regarding in-pharmacy HIV testing with the implementation of several rapid HIV testing programmes where the pharmacists are in charge of the whole testing process as a part of their everyday routine. These programmes have been working in regions very different in terms of sociology, demography, HIV new diagnosis rates and availability of testing services: Castilla y León, the Basque Country and in a part of Catalonia. Castilla y León is a region of low prevalence for HIV with an HIV incidence rate of 5.0 cases per 100000 population in 2012, largely comprised of small towns far apart from each other, very sparsely populated and with an aged population. In contrast, the Basque Country is a highly industrialized region, one of the smallest in Spain in terms of surface but also one of the most densely populated with very little distance between municipalities. The regions’ new diagnosis rates (7.7 cases per 100000 population in 2012) are similar to the Spanish average (9.5 cases per 100000 population in 2012). The regions of Catalonia where the programme was implemented are mainly comprised of towns with a high presence of immigration not far from Barcelona city-the second largest city in Spain-. Regarding HIV, it reports new diagnosis rates very similar to Castilla y Leon, 4.6 cases per 100000 population in 2012. Unlike the other two regions, Castilla y León has no specific services for HIV testing, such as HIV/STI clinics. In this region, the Spanish National Healthcare System (SNHS) offers the test free of charge only through primary health centres and hospitals.

The three programmes were launched as a partnership between the Regional Ministries of Health and the Councils of Professional Associations of Pharmacists of each region. The Basque and the Catalan programmes were launched in 2009, while the one in Castilla y León started in 2011 and they have been running since then. Although data on these programmes has been previously published, [18–20], they are only partial reports dealing mostly with the early stages of operation or piloting time. This study seeks to present a more comprehensive view of all three programmes, including longer operating time and therefore more robust results.

Our aim is to evaluate the feasibility of offering in-pharmacy rapid HIV testing as part of everyday routine and its ability to promote diagnosis in three Spanish regions with different sociological profiles and also different epidemiology for HIV. We will establish a comparison between the outcomes of each programme and those of each regional surveillance system on new HIV diagnosis (RHSS), assessing whether priority population groups are being reached and its contribution to the overall new diagnoses in each region.

## Methods

**Fig 1:**
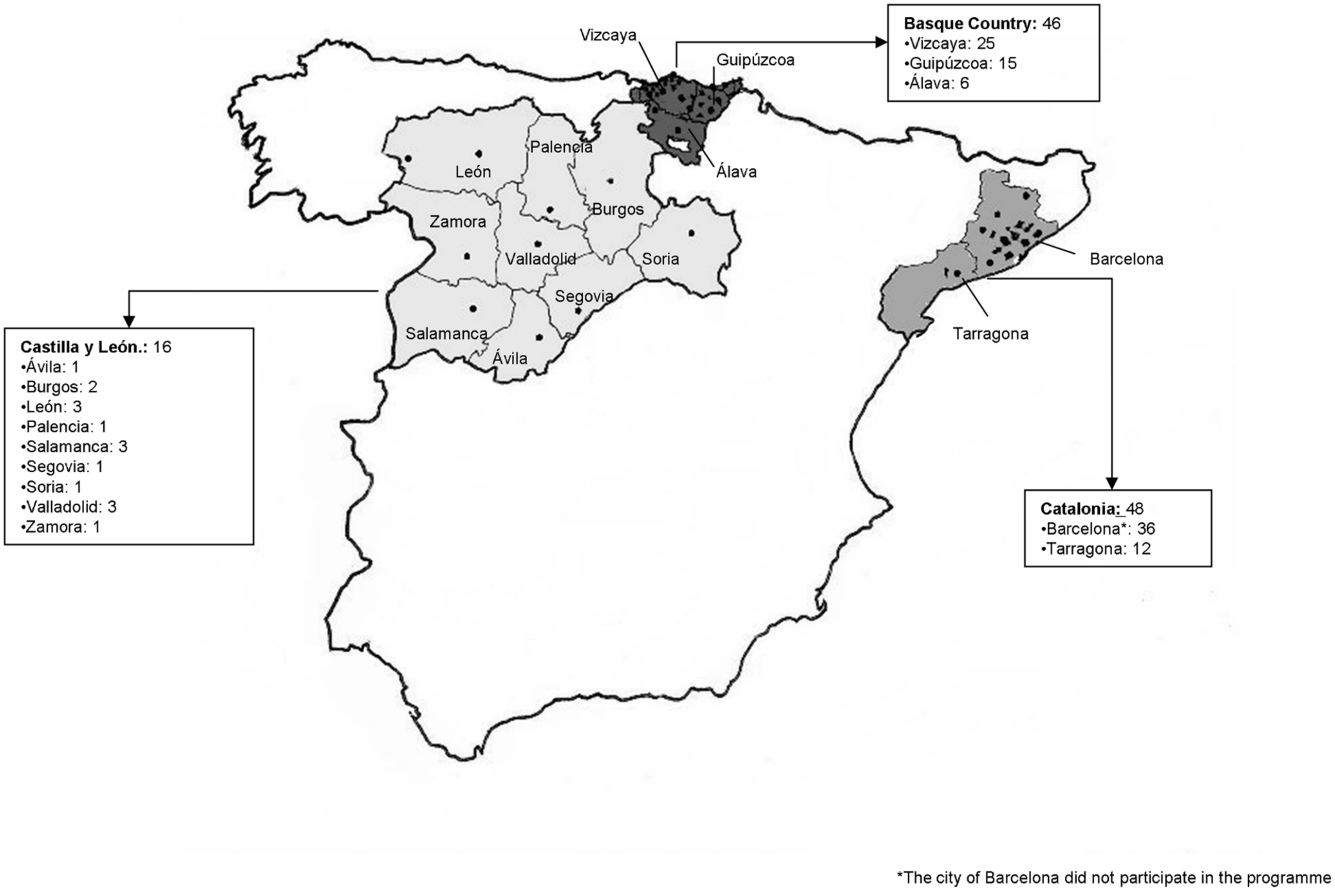
Distribution of the participating pharmacies on three rapid HIV testing programmes. Spain (2009–2013).

The data analysed here comes from a 4 year period of operation of the Basque programme (2009–2013), three years of the Catalonian (2009–2012) and two years of the one in Castilla y Leon (2011–2013). Altogether, 110 pharmacies participated in the three programmes: 16 in Castilla y León, 46 in the Basque Country and 48 in Catalonia. (Fig 1) In Castilla y León, the pharmacies were located in the provincial capital cities except one which was located in the second largest city of a province (Ponferrada). In the Basque Country, the 46 pharmacies were distributed not only in the provincial capitals, but also in other cities across the region. In the Catalan programme, 12 pharmacies were located in the two main cities of the province of Tarragona (Tarragona and Reus) and 36 neighbouring towns to Barcelona city.

Spain has a regulated pharmacy system where pharmacy chains are not allowed (the owner of a pharmacy must be a pharmacist and each pharmacist can only own one pharmacy). The number of inhabitants per pharmacy was of approximately 2200 in 2013, and the 87% of the population has a pharmacy in a range of under 250 meters.[21]. All the participant pharmacies were required to have enough space to ensure privacy, personal attention and confidentiality. The immediate response to demand and service continuity should also be guaranteed. The pharmacists received basic training in HIV testing and counselling in order to enable them to perform the test without the need of ancillary personal.

The roll-out of the programmes was advertised by press conferences and press releases. Posters and leaflets with information about each programme and data on the participating pharmacies were distributed to all pharmacies in each region. The information was also available on the official websites of the Regional Governments and the Professional Associations of Pharmacists. All the participating pharmacies exhibited a distinctive showing their participation in the initiative. Thus, all customers entering the pharmacy were informed about the possibility of testing for HIV.

The test was available during the pharmacies’ business hours for everyone over 16 years old. In Castilla y Leon and the Basque Country, the client had to pay 5 euros to cover the costs of the materials and the Administration paid the pharmacists for their time and effort. In the case of the Catalonian programme, the client paid 10 euros to the pharmacist and the Administration funded the materials (the test, the lancet, etc.). Blood collected via finger prick was tested using the Determine HIV-1/2 test (this test has a sensitivity of 100% and a specificity of over 99%). If the test was reactive, the pharmacists informed about the need of a confirmatory test as soon as possible and provided information about the reference centre and NGOs for social and psychological support. More in-depth information about the testing process and the confirmation pathways on each programme has been published elsewhere.[18–20]

Information on sociodemographic data, risk behaviours, HIV testing history and reasons for performing the test was collected from each participant through a paper questionnaire as part of the everyday routine and programme evaluation. The Castilla y Leon and the Basque programme shared very similar questionnaires. The one used in Catalonia was simpler and it was changed at midterm. Thus, not all variables were available since the beginning. The whole testing process took about 30 minutes in the case of a negative test. When the test was reactive, the time spent depended on the needs of each person.

The three programmes were launched as a partnership between the Regional Ministries of Health and the Councils of Professional Associations of Pharmacists of each region, granting ethical and legal approval and following closely the Spanish data protection law. All participants signed a written informed consent. Identifiers for both participating individuals and pharmacies were not recorded within the questionnaires. Thus, all the datasets were completely anonymous. The analysis executed using individual records were performed at regional level and in the context of public health responsibility. For the current study only aggregated data from each programme were used. For all these reasons, approval by the Ethics Committee was not required.

A descriptive analysis of the participants’ characteristics was performed stratifying the results by gender/sexual behaviour: men who have sex with men (MSM), men who have sex with women (MSW) and women. In the event of men who did not provide enough information to classify them in this manner, they were included only within the total of participants.

The results of each programme were compared with those of the regional surveillance system on new diagnosis from each region: the autonomous community of Castilla y Leon, the Basque Country and the provinces of Tarragona and Barcelona excluding results from the city of Barcelona. For this comparison, we took into account data from the years in which the programme was implemented. Statistical significance was analysed using the χ2 test for categorical variables or the Fisher's exact test when indicated. The precision of the estimates for HIV prevalence were analysed by calculating the 95% confidence interval (95%CI).

## Results

Altogether, 24151 people were tested in the three programmes and 226 had a reactive test result. The main characteristics of the participants and of those with a reactive result can be seen in Table 1.

**Table 1:** Characteristics of participants and those with a reactive result in three in-pharmacy rapid HIV testing programmes. Spain.

	Total				Basque Country (2009–2013)				Barcelona and Tarragona^1^ (2009–2012)				Castilla y León (2011–2013)			
	Total	Men (17628)^2^		Women	Total	Men (9949)^3^		Women	Total	Men (5404) ^4^		Women	Total	Men (2275) ^5^		Women
		MSM	MSW			MSM	MSW			MSM	MSW			MSM	MSW	
**PARTICIPANTS**																
**N**	24151	3482	11046	6523	13636	2086	6252	3687	7446	875	3239	2042	3069	521	1555	794
**(% row)**	(100.0)	(14.4)	(45.7)	(27.0)	(100.0)	(15.3)	(45.8)	(27.0)	(100.0)	(11.8)	(43.5)	(27.4)	(100.0)	(17.0)	(50.7)	(25.9)
	*Colum %*				*Colum %*				*Colum %*				*Colum %*			
**Age <30**	34,6	40,7	28,3	42,1	31,3	36,9	25,4	39,7	35,7	41,0	31,1	42,9	42,7	55,6	34,5	51,9
**Place of birth**																
Spain	90,4	90,4	92,3	87,0	92,0	91,5	93,6	88,6	88,6	86,9	88,9	85,8	91,2	92,0	94,0	83,8
Latin-America	5,2	5,9	3,4	7,9	4,5	5,8	2,5	8,0	5,9	6,5	5,5	7,9	4,2	5,1	2,8	7,3
Sub-Saharah Africa	0,5	0,1	0,7	0,6	0,6	0,1	0,8	0,7	0,5	0,0	0,7	0,6	0,1	0,0	0,3	0,0
Others	3,9	3,7	3,6	4,6	2,9	2,6	3,1	2,8	5	6,6	4,9	5,7	4,5	2,9	3,0	8,8
**HIV testing history** ^6^																
Never tested previously	52,0	31,8	53,7	60,4	49,4	28,0	50,4	57,6	60,6	39,4	62,4	68,7	57,1	40,8	58,1	64,5
More than 1 year	21,1	26,9	19,7	20,2	26,7	33,8	25,5	25,3					18,3	21,5	17,0	18,9
12 months or less	20,4	32,7	20,0	14,2	24,0	38,3	24,1	17,1					24,7	37,8	24,9	16,6
**REACTIVE TEST**																
**N**	226	118	51	35	110	59	23	15	75	35	14	17	41	24	14	3
**Prevalence**	0,9	3,4	0,5	0,5	0,8	2,8	0,4	0,4	1,0	4,0	0,4	0,8	1,3	4,6	0,9	0,4
(95%CI)	(0.8–1.1)	(2.8–4.0)	(0.3–0.6)	(0.4–0.7)	(0.7–1.0)	(2.1–3.6)	(0.2–0.5)	(0.2–0.6)	(0.8–1.2)	(2.6–5.4)	(0.2–0.7)	(0.4–1.3)	(0.7–1.8)	(2.7–6.5)	(0.4–1.4)	(0.1–1.1)
	*Colum %*				*Colum %*				*Colum %*				*Colum %*			
**Age <30**	33,3	41,9	20,0	23,5	25,2	30,5	17,4	7,1	39,2	52,9	14,3	29,4	47,5	54,2	30,8	66,7
**Place of birth**																
Spain	78,0	76,9	88,2	65,6	75,7	75,9	82,6	66,7	74,7	77,1	85,7	64,7	85,4	79,2	100,0	66,7
Latin-America	15,5	17,1	5,9	25,0	13,6	19,0	8,7	8,3	20,0	17,1	7,1	35,3	9,8	12,5	0,0	33,3
Sub-Saharah Africa	1,5	0,0	2,0	6,3	4,9	0,0	4,3	16,7	0,0	0,0	0,0	0,0	0,0	0,0	0,0	0,0
Others	5,0	6,0	3,9	3,1	5,8	5,2	4,3	8,3	5,3	5,8	7,1	0,0	4,9	8,3	0,0	0,0
**HIV testing history** ^6^																
Never tested previously	46,5	34,4	71,4	50,0	39,8	26,3	73,9	46,2	51,5	46,2	50,0	50,0	59,0	47,8	76,9	66,7
More than 1 year	28,9	36,6	16,7	20,8	41,7	50,9	17,4	38,5					20,5	21,7	23,1	0,0
12 months or less	15,7	21,5	4,8	12,5	18,4	22,8	8,7	15,4					20,5	30,4	0,0	33,3

^1^ Comprising main cities in the provinces of Tarragona and Barcelona but no Barcelona city.

^2^ 3100 men could not be classified according to their sexual behaviour. Their responses are included within the total. 22 tested positive.

^3^ 1611 men could not be classified according to their sexual behaviour. Their responses are included within the total. 13 tested positive.

^4^ 1290 men could not be classified according to their sexual behaviour. Their responses are included within the total. 9 tested positive.

^5^ 199 men could not be classified according to their sexual behaviour. Their responses are included within the total. None tested positive.

^6^ At the Catalonian programme, this question was available in que questionnaire only for 3287 participants and 36 positives.

Percentages calculated on contestants. MSM: Men who have sex with men; MSW: Men who have sex with women.

Approximately 46.0% of those who underwent testing were MSW whereas 52.2% of those with a reactive result were MSM. The proportion of Latin Americans was three times higher among those with a reactive result (15.5%) than among the total of participants (5.2%) (p<0.0001). Both the attendees and those with a reactive result in the programme from Castilla y León were younger than those in the other programmes (p<0.0001 when comparing with the other participants and p = 0.02 when comparing with the others with a reactive result).

The proportion of those with a reactive result who did not have a previous HIV test ranged from 59% in Castilla y Leon and 39.8% in the Basque programme. (p = 0.09) In these two programmes, the proportion of MSW who were tested for the first time in the programme was higher in the case of those who tested positive than in the total MSW attendees.

The prevalence within the Spanish was of 0.8% (95%CI: 0.7–1.0) and of 2.2 (95%CI: 1.6–2.9) within foreigners.

Regarding their contribution to the new diagnoses in each region during the study period, the Basque country in-pharmacy testing programme was able to detect the 12.7% of all the new HIV diagnoses in the Basque region between 2009 and 2013. This proportion was of 8.7% in the case of the programme conducted in Tarragona and Barcelona (conducted between 2009 and 2012) and of 10.3% in the case of Castilla y León, (conducted between 2011 and 2013). In total, 10.6% of new HIV diagnoses reported in the period of operation of the programmes are attributable to pharmacies. (Table 2)

**Table 2:** Comparison between the regional HIV surveillance systems on new diagnoses (RHSS) and three in-pharmacy rapid HIV testing programmes.

	Total		Basque Country (2009–2013)		Barcelona and Tarragona^1^		Castilla y Leon	
	In-pharmacy testing	RHSS	In-pharmacy testing	RHSS-Basq	In-pharmacy testing (2009–2012)	RHSS-Cat^2^ (2009–2012)	In-pharmacy testing (2011–2013)	RHSS-CyL (2011–2013)
Number of new diagnoses	226	2126	110	864	75	863	41	399
% of new diagnoses reported by the RHSS detected by the pharmacies	10,6		12,7		8,7		10,3	
% by transmission categories								
Injecting drug users	2,2	6,8	0,9	6,3	5,3	8,2	0,0	5,0
Heterosexual contact	36,7	39,2	33,6	39,9	38,7	37,1	41,5	42,1
Women	14,6	16,0	12,7	17,6	21,3	15,2	7,3	14,5
Men	22,1	23,1	20,9	22,3	17,3	21,9	34,1	27,6
Men who have sex with men	52,2	42,0	53,6	43,3	46,7	40,7	58,5	41,9
Others/Unknown	8,8	12,1	11,8	10,5	9,3	14,0	0,0	11,3

^1^ Comprising main cities from the provinces of Tarragona and Barcelona but no Barcelona city.

^2^ It includes data only from the territory in which the in-pharmacy programme takes place.

All three in-pharmacy testing programmes diagnosed a higher proportion of MSM than that reported by their regional surveillance systems. The highest difference was found in Castilla y Leon, where the 58.5% of those diagnosed by the pharmacies were MSM vs. the 41.9% reported by the RHSS-CyL. The Catalonian programme also found a higher proportion of women (21.3%) than the reported by its regional surveillance system (15.2%).

## Discussion

This is the first study assessing in-pharmacy HIV testing programmes performed as part of everyday routine in the pharmacy by the pharmacist, without being a pilot or a campaign. The main users of these programmes have been Spanish heterosexual men, aged 30 or more and who had no previous HIV tests. As expected, the group most affected by the epidemic, MSM, constituted the majority of the diagnosed individuals, with a high percentage of young and untested individuals. But the prevalence found among women and heterosexual men was also high. Altogether, these programmes have contributed with 11% of the total new diagnoses reported in the three regions.

All the initiatives and pilot studies that we have found on in-pharmacy HIV testing were constrained by time and scope.[14–17] Altogether, they tested less than 5000 people and raised concerns, especially regarding its long term sustainability and the pharmacist's ability to deal with the counselling in an already time-limited practice setting. [10–13] The three programmes presented here have been working between 3 and 5 years and have tested over twenty four thousand people, using regular pharmacies without any hard to achieve requirements and without needing ancillary personal. The participating pharmacists stated that the provision of this service is an added value from the professional point of view and that it improves their position as health agents with a major role in the continuum of patient care [22–26]. These arguments, leads us to say that in-pharmacy testing by pharmacists is perfectly feasible, it does not generate an unmanageable load of work for professionals and it can be seamlessly integrated into the set of services that pharmacies offer to its customers.

As one could expected, the majority of the diagnosed individuals were MSM, the group most affected by the epidemic.[27] The prevalence found within this group is very similar to those found by other programmes not aimed at high risk populations [28] and lower than those reported by programmes specifically aimed at MSM. [29,30] On the other hand, pharmacies have proven to be a very valuable option for heterosexual population, confirming what was found on preliminary evaluations. [18–20] MSW is one of the most difficult groups to reach for early diagnosis. [27] Testing services designed for most-at-risk populations might not be appealing for them and some initiatives that do aim at them have shown relatively low outcomes.[31] Also, although the global percentage of previously untested MSW is within expected, [32] the proportion of MSW with a reactive result who lack from a previous HIV test (71.4%) is quite surprising. The availability of the test in pharmacies may help to increase the coverage within this population since it offers the chance of getting tested by a health professional, without the requirement on an appointment, with immediate results and in an environment that does not imply any necessary relationship with HIV.

It is also worth to highlight the prevalence found in MSW and women (0.5% in both cases), higher than what found by other programmes not aimed at high risk populations. [28,33] Actually, this prevalence is very similar to that reported by a sentinel surveillance network of very low threshold HIV/STI clinics located in 19 medium and large size cities through Spain (0.5 for women and 0.7 for MSW) that is considered to be serving very high risk populations.[34] Although the proportion of women getting tested at these programmes is very similar across the three of them, the one in Catalonia shows a prevalence in women that almost doubles the one found by the other two. Perhaps the possibility of taking the test at pharmacies in this region is attracting women at risk who may not being reached by other initiatives.

Of the three programmes, the one in Castilla y León presented the highest prevalence. Castilla y León is a region without specific services for testing for HIV and where a significant percentage of its population lives in rural areas. This suggests that in-pharmacy testing seems especially useful for those who live in areas where access to testing services is particularly hindered by both distance and stigma. The fact that the percentage of MSM uncovered by the pharmacies was 1.4 times higher than what is reported by the regional surveillance system supports this statement. The striking percentage of MSM with a reactive result that lacked from a previous test also points towards that direction. Pharmacies may have been the first chance for them to be tested at their own convenience, in a friendly environment where no one can recognize them and without the need of disclosure or talking about their sexual life.

The impact of these programmes within the set of new diagnoses has reached 11% of the total of new diagnoses reported by the three regions. This reflects the real contribution that the three programmes have made to the promotion of HIV diagnosis, which is something rarely done.

Nevertheless, there are some limitations that must be taken into account. First, the percentage of MSW may have been overestimated. It is possible that, because of fear of disclosure and stigma, some men may have not disclosed having sex with other men. Also, we do not know how many of those with a reactive result at the pharmacy received a positive test at confirmation. Given that the anonymity is one of the key issues of the three programmes, that information is not available. That being said, we believe that linkage to care is likely to have been high since access to testing and treatment is free of charge in Spain and that the post-test counselling session emphasized the need of confirmation and the importance of early treatment.

This is the first study that provides strong evidence supporting that in-pharmacy HIV testing programmes are a valuable testing option to uncover undiagnosed infections, with more than twenty four thousand people tested and over two hundred infections diagnosed. These programmes have shown a good capacity of reaching and diagnosing previously untested populations, not only MSW- the group most affected by delayed diagnosis- but also young MSM. Overall, they have contributed with one out of 10 new diagnoses reported in the regions and they seem to be particularly suitable for those regions without large cities and specific HIV diagnostic services.
